# Role of *CYP1A1*, *CYP2D6*, and *NOS3* gene polymorphisms in idiopathic recurrent pregnancy loss in the Iranian Azeri population: A case-control study

**DOI:** 10.18502/ijrm.v20i8.11756

**Published:** 2022-09-06

**Authors:** Mahsa Yousefian, Abdolhamid Angaji, Elham Siasi, Seyed Ali Rahmani, Shamsi Abbasalizadeh Khiaban

**Affiliations:** ^1^Department of Genetics, Faculty of Biological Sciences, North Tehran Branch, Islamic Azad University, Tehran, Iran.; ^2^Department of Cell and Molecular Biology, Faculty of Biological Sciences, Kharazmi University, Tehran, Iran.; ^3^Department of Medical Genetics, School of Medicine, Tabriz University of Medical Sciences, Tabriz, Iran.; ^4^Department of Obstetrics and Gynecology, Women's Reproductive Health Research Center, Tabriz University of Medical Sciences, Tabriz, Iran.

**Keywords:** Recurrent pregnancy loss, Polymorphism, CYP1A1, CYP2D6, NOS3.

## Abstract

**Background:**

It is estimated that 1-5% of couples suffer from recurrent pregnancy loss (RPL). Recent studies have shown the effects of gene polymorphisms in RPL.

**Objective:**

The aim of this study was to evaluate 3 gene polymorphisms including rs1048943 of *CYP1A1*, rs28371725 of *CYP2D6*, and rs7830 of *NOS3* in idiopathic RPL to identify their association with RPL.

**Materials and Methods:**

Blood samples were collected from 136 women with at least 2 consecutive idiopathic miscarriages (case group) and 136 women with no history of miscarriage and at least one successful pregnancy (control group) from the Iranian Azeri population. This study was carried out between April 2018-April 2020. Amplification-refractory mutation system polymerase chain reaction was used for the rs7830, rs1048943 and rs28371725 polymorphisms in order to genotype each extracted genomic DNA sample. After that, Chi-square, Fisher's exact test and logistic regression were used to investigate whether each of these polymorphisms is associated with RPL.

**Results:**

Among these polymorphisms, only rs1048943 of *CYP1A1* showed a statistically significant association with RPL in the Iranian Azeri women studied.

**Conclusion:**

Our results suggest that *CYP1A1* gene polymorphisms might be associated with a reduced risk of RPL. Further studies in other populations and in the same population with a larger sample size, as well as functional genomics analyses such as gene expression analyses or epigenetic studies are required to validate our results.

## 1. Introduction

Pregnancy loss is defined as a loss of pregnancy before the end of the 20 ${}^{\text{th}}$ wk of gestation (1). Recurrent pregnancy loss (RPL) is defined as at least 2 consecutive miscarriages (2-4). It is estimated that 1-5% of couples suffer from RPL (5, 6). Although in 50% of cases several factors such as endocrine dysfunction, infections, environmental factors, and parental chromosomal abnormalities may cause RPL, in 50% of cases the cause is unknown (7-9).

Several studies have examined various polymorphisms of candidate genes that encode different mediators which may affect susceptibility to idiopathic RPL (4, 10, 11). A group of these genes belongs to metabolic enzymes. Genetic polymorphisms of these genes may affect the balance of phase I / phase II detoxification enzymes (12). One of these enzymes is encoded by *CYP1A1* which is located on 15q24.1 and includes 7 exons. This gene acts in a 2-step process of detoxifying toxins. In the first step, *CYP1A1* is required for activation of toxic components. These components are required for the 2 ${}^{\text{nd}}$ detoxification step. The polymorphisms of this gene can be directly linked with functional disturbance diseases and conditions like cancers and idiopathic male infertility. A recent study showed that this gene can influence normal estrogen metabolism and placental function (13). It seems that its polymorphisms may also lead to RPL.

Another gene of this pathway is *CYP2D6* which is located on 22q13.2 and includes 9 exons (14, 15). This gene is important for pharmacogenetics; it is involved in the metabolism of over 150 drugs and has been studied in individuals with suicidal thoughts or depression (16). *CYP2D6* has an important role in catalyzing the oxidation of testosterone to androstenediones (17), both of which increase during pregnancy. So, it seems that any change in this enzyme may lead to an increased risk of RPL.

There is no autonomic innervation in fetoplacental blood vessels and the regulation of vascular functions at the fetomaternal interface is mediated by endothelial nitric oxide synthase (*NOS3*), which is a vasoactive mediator (18). This gene is located on 17q36.1 and includes 26 exons. It encodes an enzyme that is important in producing vascular NO (19, 20). “NO is a gaseous molecule, which serves different physiological regulatory functions in the regulation of reproduction, such as the formation of new blood vessels, enhancement of blood supply through the maternal arteries to the placenta, regulation of the placental vessel tones, and immune protection of the fetus. All these factors are required for a successful pregnancy outcome and any disturbance in these steps may increase the risk of miscarriage" (21). In the first trimester of pregnancy, trophoblast cells express a large amount of *NOS3*, so any polymorphism in coding or noncoding regions of the gene may change its activity or expression level, which may lead to RPL (19).

The aim of this study was to determine the relationship between 3 polymorphisms of these genes and RPL in the Iranian Azeri population.

## 2. Materials and Methods

### Sampling 

This case-control study was carried out in the Rahmani genetic lab, Tabriz, Iran, during April 2018-April 2020 with 2 groups. The control group (n = 136) consisted of 19-45 yr-old women with no history of miscarriage or infertility and with at least 1 successful pregnancy and a delivery without any complications. The case group (n = 136) consisted of 16-42 yr-old women with at least 2 consecutive idiopathic miscarriages and no successful pregnancies. It should also be considered that all the women in both groups were Iranians with Azeri origin.

All women with RPL due to infections, uterine conformational abnormalities, immune disorders, hormonal abnormalities (including thyroid and prolactin disorders) and chromosomal abnormalities in themselves or their spouses were excluded from the study.

The sample size was calculated with this formula: N = (Z1-α /2) 2 p (1-p) /d2, (Z1-α /2 = 1.96, p = 0.5, d = 0.1), N = 96. So at least 96 samples were needed to perform our study but in order to control the random sampling of the errors, we increased the sample size to 136 in each group. Please refer to table I for the demographic and clinical characteristics of the cases with RPL and the control group.

### DNA extraction

5 ${}^{\text{cc}}$ peripheral blood samples were taken from each individual. Genomic DNA was isolated from a 1 ml ethylenediamine tetraacetic acid-anticoagulated peripheral blood sample using a DNA extraction kit (KBC blood DNA extraction kit, Cat. No. K1135, Tehran, Iran) according to the manufacturers' instructions. The quality of each sample was then estimated using a nanodrop for assessment of nucleic acid purity.

### Genotyping

To confirm each polymorphism in its genetic region, the minor allele frequency (MAF) should be more than 0.01. Each single nucleotide polymorphism (SNP) was chosen according to its MAF in the 1000 Genome Project. MAF has been reported as C = 0.133387/668 for rs1048943, T = 0.063498/318 for rs28371725 and T = 0.361821/1812 for rs7830 (22).

Rs1048943 is a missense coding sequence variant that causes Ile to Val change in the cyp1a1 protein; rs28371725 is a G > A intron variant which causes alternative splicing that can alter cyp2d6 function of the protein; and rs7830 is a G > T intron variant that leads to a different transcript. Detailed information about the polymorphisms including the full and abbreviated gene names, residue change and mutation type is presented in table ІІ.

Genetic polymorphisms of each participant for rs1048943 and rs28371725 were detected by allele-specific polymerase chain reaction (ARMS-PCR) and the polymorphisms for rs7830 were determined by tetra primer amplification refractory mutation system PCR (T-ARMS-PCR). The ARMS primers were designed using a WASP webpage with the address https://bioinfo.biotec.or.th/WASP/ and for T-ARMS-PCR the designs were done by the primer1 webpage with the address https://http://primer1.soton.ac.uk/. Detailed information about the primers is listed in table ІІІ and table ІV.

After their design, all the primers were evaluated with the BLAST-NCBI database and analyzed using the Oligo Analyzer software to ensure the validation of each primer. To increase the specificity of each forward primer in ARMS-PCR and each of the 2 inner primers in T-ARMS-PCR, an extra mismatch was designed in the 2 ${}^{\text{nd}}$ nucleotide for ARMS-PCR and in the third nucleotide for T-ARMS-PCR from the 3 ' end.

Amplifications were carried out in a thermal cycler (Peqlab peqSTAR 96, Erlangen, Germany) with 2 tubes per person using ARMS-PCR, (1 for the wild allele and the other for the mutant allele) with a pair of primers in each tube (wild/mutant forward and common reverse) and 1 tube for each sample using T-ARMS-PCR with 4 primers in each tube. Each tube contained 50 ml: 25 ml of 2 × PCRBIO Taq Mix Red Master Mix (Cat# PB10.13-02, London, England), 2.0 ml of each primer, 100-500 ng of genomic DNA, and up to 50 ml final volume of deionized water. After pre-denaturation at 95 C for 5 min, the PCR was carried out for 35 cycles of 30 sec at 94 C; 30 sec at 57 C for *CYP1A1*, 52 C for *CYP2D6* and 69.3 C for *NOS3*; 30 sec at 72 C and at the end of the 35 cycles, the final extension at 72 C for 2 min to complete the extension of all DNA fragments. The PCR products were analyzed in 2% agarose gel stained with gel stain and with a 50 base pairs (bp) DNA ladder as the template of measurement. To ensure the accuracy of the SNP genotypes, 15% of the samples were selected randomly and re-genotyped to verify the initial results. The results confirmed that the genotyping was valid and consistent.

**Figure 1:**
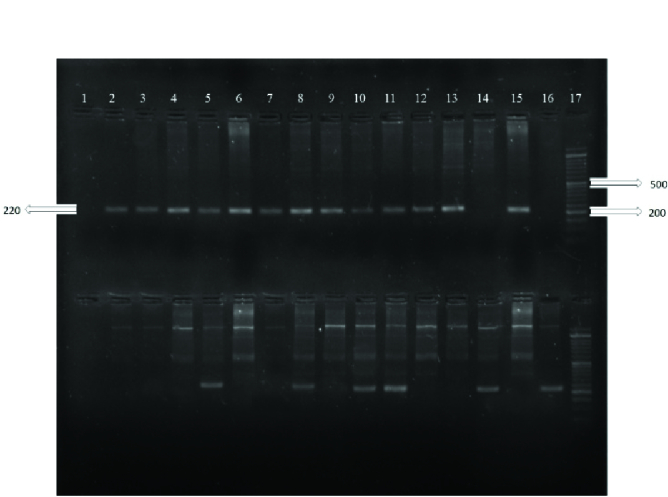
ARMS-PCR products of rs1048943. The top bands show wild alleles and the down bands show mutant alleles for each sample: Number 1 is negative control; 2, 3, 4, 6, 7, 9, 12, 13 and 15 are wild homozygotes (TT), 5, 8, 10 and 11 are heterozygotes (CT), 14 and 16 are mutant homozygotes (CC), and 17 is 50 bp DNA ladder.

**Figure 2:**
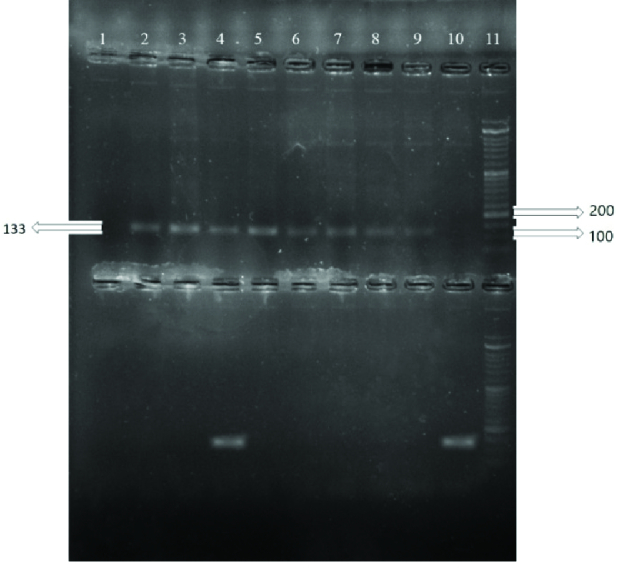
ARMS-PCR products of rs28371725. The top bands show wild alleles and the down bands show mutant alleles for each sample: Number 1 is negative control; 2, 3, 5, 6, 7, 8 and 9 are wild homozygotes (CC), 4 is heterozygote (CT), 10 is mutant homozygote (TT), and 17 is 50 bp DNA ladder.

**Figure 3:**
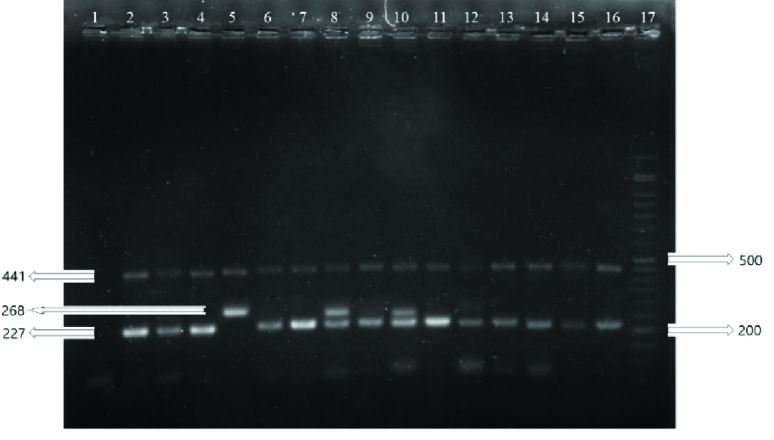
T-ARMS-PCR products of rs7830. Number 1 is negative control, 2, 3, 4, 6, 7, 9, 11, 12, 13, 14, 15 and 16 are wild homozygotes (GG), 8 and 10 are heterozygotes (TG), 5 is mutant homozygote (TT), and 17 is 50 bp DNA ladder.

The sizes of the PCR products were 220 bp and 133 bp for the rs1048943 and rs28371725 polymorphisms, respectively, and for the rs7830 polymorphism the sizes were 441, 227 and 268 bp for the common, wild and mutant products, respectively. The PCR products are shown in figures 1, 2 and 3.

**Table 1:** Demographic and clinical characteristics


**Characteristics**	**Case group**	**Control group**
Age (yr)*	28.9 ± 6.3	31.9 ± 6.3
BMI (kg/m^2^)*	27.3 ± 4.1	27.0 ± 3.9
Smoking**	0%	0%
Consanguineous marriage**	18.3%	15.4%
*Data shown as Mean ± Standard deviation. **Data shown as n (%). BMI: Body mass index		

**Table 2:** Details about the polymorphisms


**Reference SNP**	**Full gene name**	**Abbreviated gene name**	**Residue change**	**Mutation type**
**rs1048943**	Cytochrome P450 family 1 subfamily A member 1	*CYP1A1*	Ile462Phe	missense
**rs28371725**	Cytochrome P450 family 2 subfamily D member 6	*CYP2D6*	NA	intron variant
**rs7830 **	Nitric oxide synthase 3	*NOS3*	NA	intron variant
NA: Not applicable, SNP: Single nucleotide polymorphism				

**Table 3:** The ARMS-PCR primers


	**rs1048943**	**rs28371725**
**Wild forward **	GAAGTGTATCGGTGAGACAA	CCATCTGGGAAACAGTGAA
**Mutant forward**	GAAGTGTATCGGTGAGACAG	CCATCTGGGAAACAGTGAG
**Common reverse**	ACCAGACCAGGTAGACAGAG	TCCTATGTTGGAGGAGGTC
ARMS-PCR: Amplification refractory mutation system polymerase chain reaction		

**Table 4:** The T-ARMS-PCR primers


	**rs7830**
**Forward outer**	TTTTTTGAGATGGGAAGAACTTGGGTCC
**Reverse outer**	CTACTATGTACCAGGCACCAGGAAGCCA
**Forward inner**	ATGACATTGAGAGCAAAGGTGAGGCTAGG
**Reverse inner**	ACTCCCTTCAGGCAGTCCTTTAGCCA
T-ARMS-PCR: Tetra amplification refractory mutation system polymerase chain reaction	

### Ethical considerations

The Islamic Azad University, Tabriz Branch Ethics Committee, Tabriz, Iran approved this study (Code: IR.IAU.TABRIZ.REC.1398.022). All the participants completed a written informed consent form. The methods were performed in accordance with the ethical principles, national norms, standards, relevant guidelines, and regulations for conducting medical research in Iran.

### Statistical analysis

Central tendency and dispersion of the demographic data related to the clinical characteristics of our case and control groups were examined by descriptive statistics such as mean and standard deviation.

Fisher's exact test was used to compare the case and control groups. A p < 0.05 was considered statistically significant. For the SNPs with a p < 0.05, the Hardy-Weinberg equilibrium analysis was performed by using the Chi-square test and Fisher's exact test (for multiplicative and additive models of rs1048943) to compare the observed and expected genotype frequencies in both the case and control groups to determine the model of association. It was found that the polymorphisms of all of the SNPs followed the multiplicative model. The additive model which is independent of the Hardy-Weinberg equilibrium was evaluated too. To show the effect of these polymorphisms on RPL, odds ratios (OR) with a 95% confidence interval (95% CI) were calculated using logistic regression, Chi-square test and Fisher's exact test (for multiplicative and additive models of rs1048943) in the Statistical Package for the Social Sciences (SPSS), version 26 (SPSS Inc., Chicago, Illinois, USA).

## 3. Results

The mean age of the women in the control group was 31.9 ± 6.3 yr (range 19-45) and 28.9 ± 6.3 (range 16-42) in the case group. Three candidate polymorphisms were genotyped and analyzed during our study. A comparison of the frequencies of the polymorphisms in the case and control groups are shown in table V. According to the results, rs28372725 of *CYP2D6 *(p= 0.75) and rs7830 of *NOS3* (p = 0.46)did not show any statistically significant difference between the case and control groups and for this reason no more statistical steps were followed for these 2 SNPs. Regression analyses for rs1048943 of *CYP1A1* (p < 0.001)under the multiplicative model and additive model were carried out and the results are shown in table VI. According to our findings, rs1048943 of *CYP1A1* was the only polymorphism that was statistically associated with RPL in both multiplicative and additive models. As shown in table VI, the association of rs1048943 with RPL (p < 0.001, OR [95% CI] = 0.342 [0.221-0.531]) was observed under the multiplicative genetic model. In this SNP, TT is the wild genotype, so it was considered as the reference genotype. According to the additive genetic model, CC (OR [95% CI] = 0.111 [0.032-0.388], p < 0.001) and CT (OR [95% CI] = 0.463 [0.265-0.807], p = 0.01) of this SNP were associated with RPL using TT as the reference genotype.

Although the genotype frequencies of TT and CT in rs28372725 of *CYP2D6* were higher in the controls than in the cases, we failed to find any statistically significant association of these with RPL. Finally, rs7830 of *NOS3* did not show any significant association with RPL either.

**Table 5:** Genotype frequency of the polymorphisms in both groups


**SNP**		**Case n (%)**	**Control n (%)**	**P-value**
**rs1048943**				
	**CC**	3 (2.2)	19 (14.0)	
	**CT**	29 (21.3)	44 (32.4)	
	**TT**	104 (76.5)	73 (53.7)	< 0.001
**rs28371725 **				
	**TT**	1 (0.7)	2 (1.5)		
	**CT**	10 (7.4)	12 (8.8)	
	**CC**	125 (91.9)	122 (89.7)	0.75
**rs7830**				
	**TT**	3 (2.2)	4 (2.9)		
	**TG**	31 (22.8)	23 (16.9)	
	**GG**	102 (75.0)	109 (80.1)	0.46
SNP: Single nucleotide polymorphism. Calculated using Fisher's exact test				

**Table 6:** Multiplicative and additive genetic models


**SNP**	**Multiplicative model**	**Additive model**		
**rs1048943**	C vs. T	CC vs. TT	CT vs. TT	TT
**OR**	0.342	0.111	0.463	1 (reference)
**95% CI**	0.221-0.531	0.032-0.388	0.265-0.807	
**P-value**	< 0.001	< 0.001	0.01	
SNP: Single nucleotide polymorphism. Calculated using Fisher's exact test for the multiplicative and additive model (CC vs. TT) and Chi-square test for the additive model (CT vs. TT)				

## 4. Discussion

*CYP1A1* plays an important role in the oxidation of polycyclic aromatic hydrocarbons like benzopyrene and polychlorinated biphenyls. These substances are unusual environmental toxicants (13). The role of this enzyme in activating carcinogens in cancers has been shown in previous studies (23-25). A recent study also showed that placental *CYP1A1* mRNA levels were higher in women with RPL (26). We also know that *CYP1A1* can affect the metabolism of estrogen and normal functions of the placenta (13). In fact, this enzyme participates in the metabolism of estrogen by catalyzing the 2-hydroxylation of stradiol (27) and can convert endogenous estrogens into more hydrophilic compounds (28). This enzyme also has an important role in the metabolism of enzymatic xenobiotics that are specifically induced in women exposed to tobacco smoke. These xenobiotics may transfer through the placenta to the fetus and cause toxicity in the fetus or may affect the expression or production of placental hormones and change the function of proteins (29). Thus, it seems that it could be a good candidate gene in relation to RPL.

One of its polymorphisms is rs1048943 A > G which is located in 2455 nucleotides and causes Ile > Val in the amino acid chain. This amino acid change occurs near the hem group of the protein and causes the enzyme activity to double (30). This polymorphism is a risk factor for pharyngeal, prostate, lung, oral, ovarian, bladder, colorectal, and cervical cancers (31). A protective association also was shown between this polymorphism and coronary artery disease in a study conducted with the Han population of China in 2017 (32). In our study, we found that rs1048943 was associated with a reduced risk of RPL in women of Iranian Azeri origin. However, another study done in 2014 in Russia did not show any association between this polymorphism and RPL (33). Given the varied results, studying this common polymorphism of *CYP1A1* in larger populations with different genetic backgrounds is recommended.

*CYP2D6* is active in catalyzing the oxidation of testosterone to androstenediones (17). Both of these hormones increase during pregnancy. The normal fertility process requires an adequate supply of sex hormones and the normal functioning of *CYP2D6* is needed to reach this threshold. *CYP2D6* is also an important modulator of the detoxification system, and can protect the environment of the utero-placental tissue from being affected by overwhelming oxidative stress (6). The activity of this enzyme begins to increase in the early stages of the 2 ${}^{\text{nd}}$ trimester of pregnancy and continues to increase as the pregnancy progresses. Nowadays it is also known that many commonly used drugs in pregnancy are metabolized by this enzyme. Considering the highly polymorphic nature of this gene (28), it seems that this could be a good candidate gene in RPL associated studies. This enzyme is also important in pharmacogenetics and is active in the biotransformation of more than 150 drugs including antipsychotics, antidepressants, analgesics, beta-blockers, and antiarrhythmics (16).

Rs28371725 causes an A > G change in nucleotide 2989 in intron 6 which brings about alternative splicing and so changes the structure of its protein. This structure change results in a reduction in enzyme activity (reduction of metabolizing of beta-blockers). The influence of this polymorphism has been studied in cases of early onset of severe pre-eclampsia; its therapeutic responses and the association between this polymorphism and early-onset preeclampsia were observed (34). To the best of our knowledge, ours is the first study to evaluate the effect of this SNP in RPL. Our research showed that the association between this SNP and RPL in the Iranian Azeri population was not significant. Another study which was conducted in the South of India in 2004 investigated the association between rs3892097 of this gene and RPL but it also did not find any positive or negative association between rs3892097 and RPL (35).

“*NOS3* encodes an enzyme that generates NO in endothelial cells and is involved in the regulation of vascular functions". The endometrial expression of *NOS3* reaches its peak in humans during implantation (36). NO also plays an important role in blood pressure control, cardiovascular homeostasis, the metabolism of glucose, and insulin resistance during pregnancy. A reduction of NO production may cause pregnancy-related vascular problems like RPL. Additionally, alteration in NO synthesis may cause premature labor by affecting the inflammatory response and uterine contractility regulation (37). As a vasoactive agent, NO causes vasodilation and increases the rate of nutrient supply and oxygen perfusion in the umbilical cord. So, any decrease in its rate of production may cause intraurine hypoxia, restriction in fetal growth and increased feto-placental vascular resistance (38). We also know that the inhibition of NO synthesis in pregnant rats causes hypertension, proteinuria, thrombocytopenia and fetal growth restriction that can all lead to miscarriage (39). Hence, this gene could also be a good candidate gene for RPL associated studies.

Rs7830 (G > T) is associated with 2 different genes: *NOS3* and *ATG9B*, but because it is located within a silencer motif, TGGGGAC, where a G > T change can lead to a different transcript, it seems that it influences the *NOS3* gene more. Although we did not find any association between this SNP and RPL, the association of this SNP with end-stage renal disease has been previously demonstrated (40). The association between NOS polymorphisms and renal dysfunction (41), atherosclerotic vascular diseases (42), and advanced diabetic nephropathy (43) have also been shown. A study done in 2013 in China could not find any association between this SNP and RPL (44). Another study conducted in Russia in 2019 showed a positive association between rs2070744 of the *NOS3* gene with miscarriage (20).

This research had some limitations. Firstly, we did not have any information about the women's family history of miscarriage. Secondly, although we asked about the practice of smoking, we did not have any information about other lifestyle habits like alcohol consumption that may have affected the results. Thirdly, our study was limited to only 1 origin within 1 country as we did not assess RPL cases and healthy populations from other ethnic groups or countries. Fourthly, our case and control groups were relatively small, and finally, we investigated only 1 SNP from each gene. Therefore, in future studies, it would be beneficial to use larger groups from various nations, with awareness of the family history of miscarriage and lifestyle practices, and also to investigate other SNPs from these genes.

## 5. Conclusion

Our results indicated that while rs1048943 of *CYP1A1 *wasassociated with a decreased risk of RPL in the studied population, there was no statistically significant association between rs28372725 of *CYP2D6* or rs7830 of *NOS3* and RPL in the same population. Further studies in various populations are needed to confirm whether these polymorphisms have any positive or negative associations with RPL.

##  Conflict of Interest

The authors declare that there is no conflict of interest.
